# Photoelectron Spectroscopy of the Low Energy States of SmO^+^ and GdO^+^

**DOI:** 10.1021/acs.jpca.6c04664

**Published:** 2026-08-08

**Authors:** Caitlyn Armendariz-Dollar, Jiande Han, Michael C. Heaven

**Affiliations:** Department of Chemistry, 1371 Emory University, Atlanta, Georgia 30322, United States

## Abstract

Associative ionization reactions of the type Ln + O → LnO^+^ + e^–^ (where Ln is a lanthanide) are being investigated as they may be used to transiently modify local electron densities at high altitudes. The most promising elements are those for which the LnO bond dissociation energy (BDE) exceeds the LnO ionization energy (IE). Consequently, accurate measurements of the BDE’s and IE’s are needed for the identification of exothermic associative ionization reactions. Sounding rocket experiments have been carried out for releases of thermally vaporized Sm. Emission spectroscopy was used as a diagnostic for the Sm + O reaction. It was hoped that the degree of ionization could be deduced from emission spectra, but the analysis has been inconclusive due to the lack of spectroscopic data for SmO^+^. In the present study we have mapped the low energy states of SmO^+^ using pulsed field ionization—zero kinetic energy (PFI-ZEKE) photoelectron spectroscopy. The data obtained are suitable for use in the analyses of the overlapped SmO/SmO^+^ spectra. The Gd + O → GdO^+^ + e^–^ reaction is known to be exothermic, but a wide range of values for the IE have been reported from both experimental observations and theoretical calculations. In the present study PFI-ZEKE measurements have been used to establish a more accurate IE (6.075 ± 0.001 eV) and the vibrational frequency for the GdO^+^ ground state.

## Graphical abstract

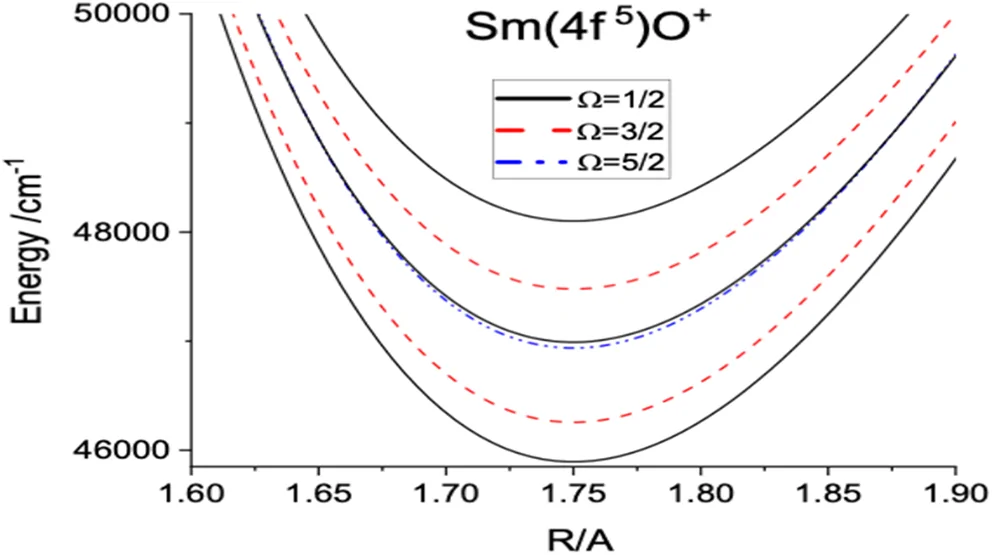

## Introduction

Gas phase associative ionization reactions of the type

1 $$\mathrm{Ln}+O\to {\mathrm{LnO}}^{+}+e^{-}$$

are known to be exothermic for several lanthanide (Ln) elements. This occurs when the LnO bond dissociation energy (BDE) exceeds the ionization energy (IE). As discussed by Schofield,^1^ the resulting LnO^+^ ions are expected to be relatively long-lived. Hence, in addition to the intrinsic interest in associative ionization processes, there are some applications where reaction 1 may be used as a source of electrons. Of relevance to the present study, communication with orbiting satellites and space vehicles is subject to interference resulting from the fluctuating electron densities encountered in the upper atmosphere.^2^ These uncontrolled signal modifications are also detrimental to targeting and GPS systems. It has been proposed that deliberate modification of high-altitude electron densities might be used to achieve temporary control of radio wave and microwave propagation.^3–7^ The associative ionization reactions with Ln = Sm or Nd are being evaluated for their ability to transiently increase the electron density via controlled, high-altitude release of the atomic metal vapors. Sounding rocket experiments have been conducted using both Sm and Nd (referred to as metal oxide space cloud (MOSC)) measurements.^4–6^ The experiments conducted with Sm provided the most detailed information, which included recordings of the visible range emission spectra.^3–5,7^ The time evolution of these spectra has been interpreted as showing the conversion of Sm to SmO and SmO^+^ via reactions with the ambient O atoms. The degree of ionization achieved in the Sm release experiments has been estimated with broad uncertainties. Modeling of the Sm MOSC data indicated that the number density of SmO^+^ ions generated was lower than that expected based on the original estimates of the exothermicity of the associative ionization reaction (approximately −0.3 eV).^4,5,8^ Subsequent remeasurements^9–11^ of the SmO BDE and IE have shown that the reaction is endothermic by 0.048 eV, which partly accounts for the lower-than-expected ion yield. In principle, the uncertainty for the ion yield could be reduced through analyses of the MOSC spectra. However, the lack of spectroscopic data for SmO^+^ has prevented this method of quantitation.

It is established that the associative ionization reaction of Nd + O is rapid^6^ and exothermic by 1.76 ± 0.10 eV.^12^ MOSC experiments have been carried out using Nd but they did not include spectroscopic measurements.^3^ Surprisingly, the movement of the cloud followed the neutral winds, rather than the local magnetic field. This suggested that the degree of ionization was low.^13^ To facilitate future MOSC field trials with Nd + O, VanGundy et al.^14^ used high resolution photoelectron spectroscopy to characterize the ground state and low-lying excited states of NdO^+^.

Gd + O is another example of an associative ionization reaction that is clearly exothermic with an enthalpy of −1.54 ± 0.10 eV.^15^ This value was obtained via accurate measurements for the BDE of GdO^+^ and the IE of Gd. High-quality spectroscopic data have been reported for GdO^16–20^ but, there are no gas phase spectroscopic data for GdO^+^ or reported high altitude release experiments for Gd.

Fundamental scientific interest in the lanthanide oxides has stimulated systematic computational studies of the LnO and LnO^+^ series.^21–23^ Electronic structure calculations for LnO/LnO^+^ species are challenging due to the presence of strong relativistic effects and partially occupied 4f and/or 5d orbitals. Consequently, accurate spectroscopic data for LnO/LnO^+^ establish benchmarks for the testing and development of basis sets and relativistic models. Most recently, Nakamura et al.^22^ have carried out extensive calculations for the complete set of LnO/LnO^+^ and LnS/LnS^+^ diatoms. They used a spin–orbit multiconfiguration quasi-degenerate second-order perturbation theory (SO-MCQDPT2) model with relativistic basis functions to predict bond lengths and excited state energies. BDE’s were calculated using a renormalized coupled-cluster method (CR-CC­(2,3)). Where experimental data is available, there is good overall agreement between the measurements and the computed values. Gas phase spectroscopic data exist for most of the neutral LnO molecules, but are limited for the cations NdO^+14^ and DyO^+^.^24,25^ Vibrational frequencies for LnO^+^ ions trapped in cryogenic Ar matrices were determined by Wilson and Andrews (Ln = Ce, Pr, Nd, Sm, Eu and Gd).^26^

In the present study we have used high-resolution photoelectron spectroscopy techniques to characterize the ground and low-lying electronic states of SmO^+^ and GdO^+^ in the gas phase. Prior to the work of Nakamura et al.,^22^ computational studies of SmO and SmO^+^ had been carried out using density functional theory (DFT),^23^ ab initio methods,^8^ and ligand field theory (LFT).^27^ The energies and Ω assignments (where Ω is the unsigned projection of the electronic angular momentum along the internuclear axis) of the excited states of SmO within the first 2200 cm^–1^ were in reasonable agreement with the experimental data for both the SO-MCQDPT2 and LFT calculations. The most accurate value for the IE was obtained by Nakamura et al.,^22^ with an error of 0.010 eV compared to the spectroscopically determined value.^9^ Ground state properties of SmO^+^ have been calculated and Nakamura et al.^22^ reported predictions for the electronically excited states.

In their discussion of the thermodynamics of Gd + O, Demireva et al.^15^ noted that a wide range of values have been reported for the IE of GdO. Experimental estimates range from 5.75 to 6.7 eV. From a critical assessment of these data, Demireva et al.^15^ recommend an IE of 5.82 ± 0.16 eV. This was based on a thermodynamic cycle using the IE of Gd (6.150 eV^28^), the BDE of GdO^+^ (7.69 ± 0.10 eV^15^) and the BDE for neutral GdO (7.36 ± 0.13 eV^29^). Recent computational estimates of the BDE include values of 6.68 (DFT),^23^ 6.996 (CR-CC­(2,3))^22^ and 7.19 (CCSD­(T))^30^ eV. To narrow the energy ranges for the IE and BDE we provide a more accurate value of the IE of GdO and vibrational intervals for the X(1)^8^Σ^–^ ground state of GdO^+^.

## Experimental Section

The equipment and techniques used for these measurements have been described previously.^31,32^ Gas phase SmO and GdO were generated using pulsed laser ablation of the metals in a supersonic expansion source. A pulsed Nd/YAG laser operating at 1064 nm was used for ablation of a samarium (99.9% purity, American Elements) or gadolinium (99.9% purity, ThermoScientific) rod. The expansion was driven by a carrier gas consisting of 1% O_2_ in He. The source pressure was typically 60 psia. Spectra were recorded using laser-induced fluorescence (LIF), resonantly enhanced two-photon ionization (RE2PI) and pulsed field ionization—zero kinetic energy (PFI-ZEKE) photoelectron techniques. LIF spectra were observed in the source vacuum chamber using a single tunable dye laser. RE2PI and PFI-ZEKE data were recorded in a differentially pumped analysis chamber that housed a time-of-flight mass spectrometer (TOF-MS) for cation detection, and a microchannel plate for photoelectron detection. Two tunable pulsed dye lasers were used for RE2PI and PFI-ZEKE measurements (Lambda Physik ScanMate Pro and Continuum ND6000). The output from the ND6000 was frequency doubled to provide tunable near UV light. The laser beams were counter propagated across the analysis chamber and overlapped in both space and time. Wavelength calibration of the fundamentals from the dye lasers was provided by a wavemeter (Bristol Instruments model 871).

The ionization-detected measurements reported here involved two-step photoexcitation. The first photon promoted the molecules to an intermediate electronically excited state. For RE2PI the second photon causes ionization provided the sum of the photon energies exceeds the IE. Mass resolved detection of the resulting cations was accomplished using the TOF-MS. For PFI-ZEKE measurements the second photon excites the molecule to long-lived high-*n* Rydberg states that exist just below each eigenstate of the LnO^+^ ion. Delayed pulsed field ionization is then used to eject the electrons from the Rydberg states. The delay discriminates against the detection of promptly emitted electrons. For the present measurements an electric field pulse of 1.4 V/cm was applied 2 μs after the laser excitation pulses.

Both Sm and Gd have multiple stable isotopes with significant natural abundancies. However, the isotopologue splittings for SmO and GdO were below the instrumental resolution. Consequently, the data reported here are weighted isotopologue averages.

## Notation

The convention used in the following to label the states of LnO^+^ molecules considers two energy ranges. The low-energy states (typically below 10000 cm^–1^) are labeled as (*n*)­Ω for the *n*-th state of angular momentum projection Ω (ascending energy order). States above 10000 cm^–1^ are labeled as [*T*_0_/1000] Ω where *T*_0_ is the term energy in cm^–1^ units.

## Results and Analysis

### SmO

As an accurate IE for SmO was already known,^9^ we could move directly to the PFI-ZEKE characterization of the low-energy states of SmO^+^. Most of the data were recorded using excitation via the [16.59]­1-X(1)­0^–^ 0–0 band.^9,33,34^ The first photon was set to excite the rotational Q-branch feature of this band at 16586 cm^–1^. Figure 1 shows the low-energy region of the PFI-ZEKE spectrum. Vibronic progressions for the ground state and the first four electronically excited states were evident. The *v* = 0 level of a fifth excited state was also present.

**Figure 1:**
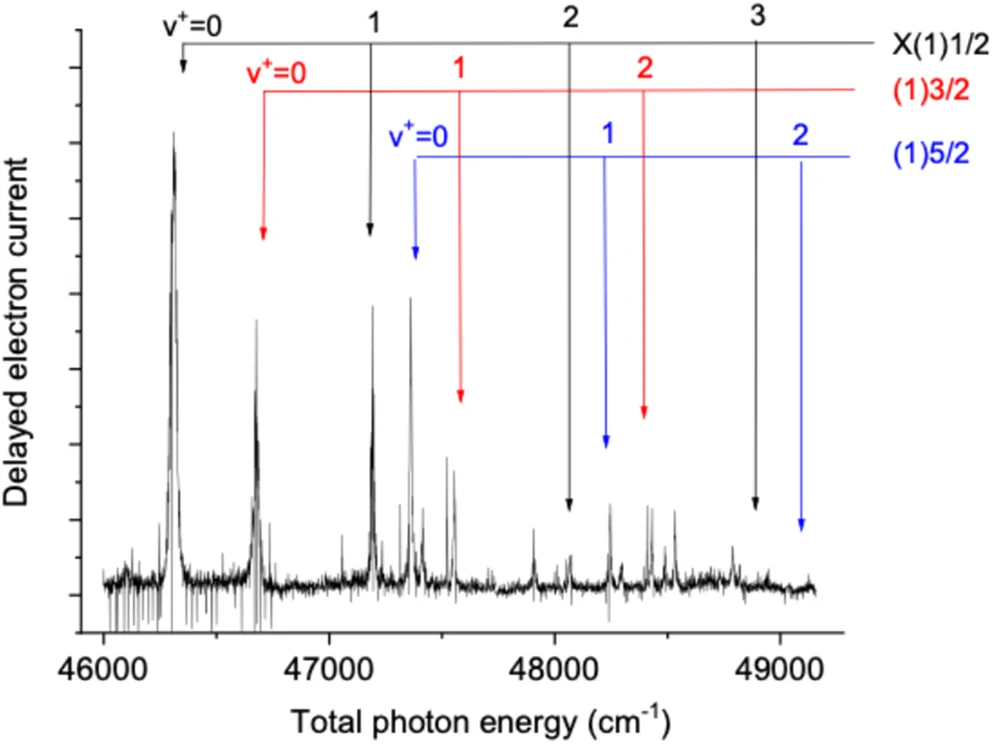
PFI-ZEKE spectrum of SmO^+^ recorded using first photon excitation of the SmO [16.59]­1-X(1)­0^–^ 0–0 band at 16586 cm^–1^.

To estimate state energies from spectra such as the example shown in Figure 1 requires some consideration of the factors that define the band intensity contours. The shape of these bands is produced by a combination of the number of rotational lines excited by the first photon and the magnitude of the pulsed electric field. Increasing the field increases the range of high-*n* Rydberg states that can be ionized, degrading the resolution by extending the red wing of the contour. The blue edge of the contour indicates the convergence of the high-*n* Ryberg levels on the eigenstate of the cation, so our analysis was focused on locating the blue edges. This has been obtained by linear extrapolation to the baseline of the steepest descent region of the blue edges. The absolute errors in these determinations are estimated to be ±10 cm^–1^. Note that there are a few unassigned peaks in Figure 1 that cannot be assigned to any of the vibronic levels of SmO^+^. These are most probably due to accidental one-color, two photon transitions of unidentified ablation products.

Table 1 lists the energies derived from Figure 1 and another spectrum obtained using a different first photon excitation for SmO. This band, centered at 15418 cm^–1^, was reported by Schmitz et al.^35^ and assigned to the [17.20]­2–(1)­1, *v*″ = 2 transition. However, using this transition for PFI-ZEKE measurements clearly demonstrated that the lower state was (1)­1 *v*″ = 0, which is 147 cm^–1^ above the X(1)­0^–^ *v*″ = 0 ground state.^33^ With this assignment correction, the IE’s derived using the two different initial excitation pathways were the same (fortuitous given the error ranges). We also note that the IE provided by these measurements differs from the value reported by Cox et al.^9^ by 11 cm^–1^, with overlapping error ranges.

**Table 1:** Energies and Vibronic Assignments for SmO^+^

SmO^+^	*E*_total_ ^a^	*E*_total_ ^b^	Δ*E* ^c^	Δ*G* ^d^	Δ*G* ^e^
X(1)1/2, *v* = 0	46,329	46,329	0	872	867.0
X(1)1/2, *v* = 1	47,201	47,196	5	872	
X(1)1/2, *v* = 2	48,073				
(1)3/2, *v* = 0	46,691	46,685	6	870	873.0
(1)3/2, *v* = 1	47,561	47,558	3	876	
(1)3/2, *v* = 2	48,437				
(1)5/2, *v* = 0	47,372	47,369	3	880	
(1)5/2, *v* = 1	48,252				
(2)1/2, *v* = 0	47,425	47,419	6	879	
(2)1/2, *v* = 1	48,304				
(2)3/2, *v* = 0	47,913	47,909	4	882	
(2)3/2, *v* = 1	48,795				
(3)1/2, *v* = 0	48,536				

a Energy (cm^–1^) of SmO^+^ state relative to the zero-point level of SmO X(1)­0^–^. These values were determined using first photon excitation of the [16.59]­1-X(1)­0^–^ 0–0 band.

b Energy (cm^–1^) of SmO^+^ state relative to the zero-point level of SmO X(1)­0^–^. These values were determined using first photon excitation of the [15.57]­2-(1)­1 band.

c Difference in state energies for columns 2 and 3.

d Vibrational intervals for the energies listed in column 2.

e Vibrational intervals for the energies listed in column 3.

### GdO

The [18.47]­4–X(1)^9^Σ transition at 18472 cm^–1^ was used to populate the intermediate excited state of GdO.^19^ An initial estimate for the IE was obtained with fixed wavelength excitation of the above transition, and a sweep of the second photon energy over the range where the ionization threshold was expected. The resulting photoionization efficiency curve yielded an IE of 48975 ± 30 cm^–1^, after correction for the local electric field (364 V cm^–1^) present in the mass spectrometer. Guided by this initial estimate, the PFI-ZEKE technique was applied to improve the accuracy of the IE and search for excited levels of GdO^+^. Figure 2 shows the resulting spectrum. As predicted, only vibrationally excited levels of the Gd^3+^(4f^7^)O ^2–^ X^8^Σ ground state were evident. The *v* ^+^ = 0, 1, and 2 levels were observed at total photon energies of 48996 (=IE), 49880, and 50,777 cm^–1^, with errors of ±10 cm^–1^. The average vibrational interval was 890 ± 10 cm^–1^. The peak marked with an asterisk is not associated with GdO^+^.

**Figure 2:**
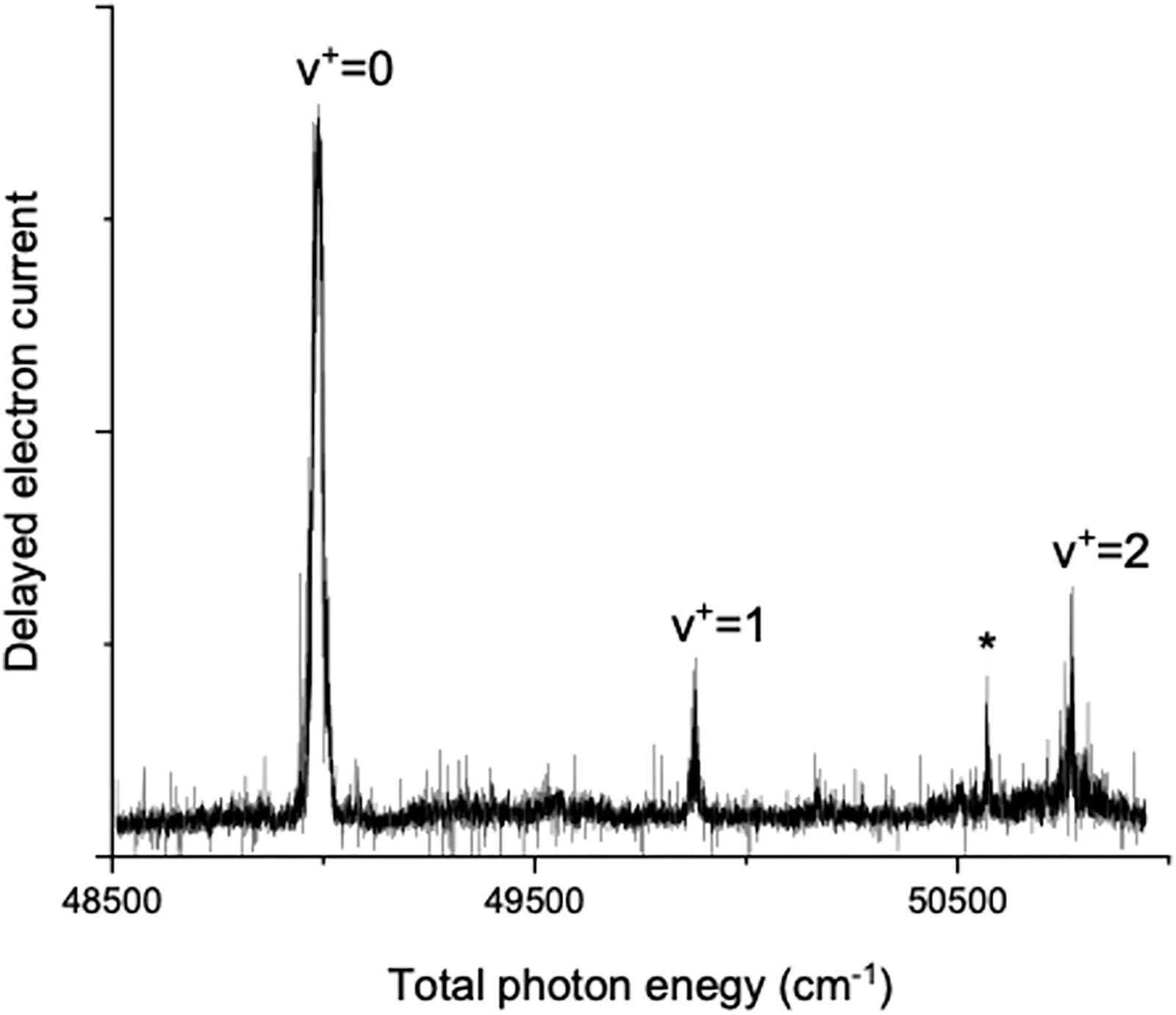
PFI-ZEKE spectrum of GdO^+^ recorded using first photon excitation of the GdO [18.47]­4-X(1)^9^Σ band at 18472 cm^–1^.

## Discussion

Electronic structure calculations for SmO predict that the ground and low-energy electronically excited states are derived from the formal Sm^2+^(4f^5^6s)­O^2–^ configuration.^22,27^ Using LFT, Carette and Hocquet^27^ predicted the energies and Ω values for more than 70 states with energies below 11000 cm^–1^. In agreement with experimental data, the ground state was predicted to be X(1)­0^–^. Nakamura et al.^22^ predicted energies and Ω values for SmO states below 10000 cm^–1^. Experimental and computational results are listed for comparison in Table 2. Here it can be seen that the calculations reproduced the observed state ordering and provided energies that were reasonably close to the experimental data.

**Table 2:** Observed and Calculated Energies^a^ for the Low-Lying States of SmO

state	*E*(obs)^33^	*E*(LFT)^27^	*E*(SO-MCQDPT2)^22^
X(1)0^–^	0.0	0	0
(1)1	147.0	115	116
(1)2	566.8	498	454
(2)0^+^	582.2	640	494
(2)1	879.3	873	738
(1)3	1280.5	1189	1058
(3)0^+^	1546.4	1359	1361
(2)2	1604.2	1517	1385
(3)1	1661.4	1472	1387
(3)2	2239.9	1998	1898
(1)4	2286.6	2226	1987

a cm^–1^ units.

This level of agreement indicates that the SO-MCQDPT2 results for SmO^+^ are accurate enough to yield electronic state assignments. As a further test we also made a brief computational study of SmO^+^ using the Molpro 2015.1 electronic structure software.^36^ Single point energy calculations were carried out at the equilibrium internuclear distance of 1.77 Å. The first stage was a state-averaged CASSCF treatment that included 5, 7, 7, and 4 sextet states of A_1_, B_1_, B_2_ and A_2_ symmetry (Molpro adopts C_2v_ symmetry for heteronuclear diatomics). This was followed by a configuration interaction (CI) calculation that was restricted to the reference configurations. Scalar relativistic effects were included using the third order Douglas Kroll method. Spin–orbit coupling was implemented using the Briet-Pauli Hamiltonian (SO–CI). The all-electron ANO-RCC-MB and augmented valence triple-ζ basis sets were used for Sm and O, respectively. Table 3 presents the measured and calculated state energies for SmO^+^, where it is evident that the SO-MCQDPT2 and SO–CI models gave results of similar quality.

**Table 3:** Observed and Calculated Energies^a^ for the Low-Lying States of SmO^+^

state	*E*(obs)	*E*(ref 22)	*E*((SO–CI)
X(1)1/2	0	0	0
(1)3/2	362	249	305
(1)5/2	1043	844	927
(2)1/2	1096	886	954
(2)3/2	1584	1431	1403
(3)1/2	2207	2041	2034

a cm^–1^ units.

Prior to the determination of an accurate value for the IE of SmO, computational values were obtained using CASSCF-CASSI (5.58 eV) and DFT (6.160 eV) methods. The most recent computational value of Nakamura et al.,^22^ 5.7528 eV, is impressively close to the measured value of 5.7427 ± 0.0006 eV.^9^

The SmO^+^ vibrational intervals listed in Table 1 were not sufficiently accurate for determination of the anharmonicity constants and they were not dependent on the electronic state, within the statistical uncertainties. The average vibrational interval was 876 ± 5 cm^–1^. This characteristic value reflects the fact that permutation of the electrons in the 5f orbitals has little effect on the bond strength. The low-energy states of neutral SmO similarly exhibit a characteristic frequency of 820 ± 5 cm^–1^ (ref 35).

The formal configuration Gd^3+^(4f^7^)­O^2–^ of the cation gives rise to the X(1)^8^Σ^–^ ground state. Due to the stability of the half-filled 4f^7^ subshell, electronically excited states were not observed nor expected in the energy range that was examined. The IE obtained from the PFI-ZEKE measurements, 6.075 eV, validates the calculations of Nakamura et al.^22^ and Smirnov and Solomonik,^30^ who obtained predictions of 6.022 and 6.089 eV, respectively. The revised IE for GdO is 0.25 eV higher than the previously recommended estimate of 5.82 eV. This change has no effect on the exothermicity of the Gd + O associative ionization reaction as the most accurate determination^15^ was based on reliable values for the IE of Gd and the BDE of GdO^+^. The revised IE does increase the BDE of neutral GdO to 7.62 ± 0.10 eV. The difference between the BDE’s of GdO and GdO^+^ is just 0.08 eV.

For both SmO and GdO the BDE’s are almost unchanged on ionization via removal of the 6s electron. This confirms the expectation that the 6s orbital is essentially nonbonding. Despite this circumstance there is evidence that the shapes of the potential energy curves near the equilibrium distances are influenced by ionization. This is apparent in the ΔG_1/2_ vibrational intervals measured in Ar matrices and the gas phase. Table 4 presents the relevant results. Note that the vibrational intervals for the Ar matrix are systematically lower than those measured in the gas phase. This is a well-known effect of the matrix host, and the magnitude of the shifts is typical for metal oxides trapped in Ar. The clear trend illustrated by Table 4 is that the vibrational intervals experience a significant blue-shift on ionization.

**Table 4:** Ground State Vibrational Intervals for SmO/SmO^+^ and GdO/GdO^+^

molecule	Δ*G*_1/2_(Ar matrix)^a^ ^,^ ^b^	Δ*G*_1/2_(gas phase)
SmO	807.4	821.8^c^
SmO^+^	840.0	872
GdO	812.7	825.4^d^
GdO^+^	844.8	886

a cm^–1^ units.

b Wilson and Andrews.^26^

c Bujin and Linton.^33^

d Suárez and Grinfeld^20^

## Conclusions

The PFI-ZEKE spectra for SmO^+^ and GdO^+^ provide valuable benchmarks for tests of the reliability of relativistic quantum chemistry models. In particular, our results show that the SO-MCQDPT2 ^22^ and CCSDT­(Q)^30^ methods provide near quantitative predictions for the ground and low-lying electronically excited states of the ions and neutral molecules. It is noteworthy that the IE for GdO reported here (6.075 ± 0.001 eV) is 0.25 eV higher than the value of 5.82 eV obtained from a critical analysis of earlier measurements and calculations.

Our experimental results and the theoretical predictions show that the ionization of LnO species results in an increase of the characteristic vibrational frequencies of the ground and low-lying electronically excited states. Ionization of both SmO and GdO occurs by the removal of the electron from the 6s orbital. This is nominally nonbonding, but it is polarized away from the O^δ−^ ligand by electrostatic repulsion. Removal of the 6s electron reduces the repulsive force, resulting in a shorter equilibrium distance and a higher vibrational frequency. Quantitative knowledge of this effect facilitates the analyses of the Sm MOSC field measurements. Vibrational progressions of SmO^+^ can be identified using the vibrational constants reported here as the differences between the vibrational intervals of SmO^+^ and SmO are readily resolved. The patterns of low-energy electronic states of SmO^+^ and SmO are also easily differentiated. Similarly, the vibrational intervals recorded for GdO^+^ will facilitate determination of GdO/GdO^+^ ratios in spectra recorded from weakly ionized plasmas.

## References

[1] Schofield K. (2006). An Overlooked
Series of Gas Phase Diatomic Metal Oxide Ions that Are Long-lived. J. Phys. Chem. A.

[2] Kung
Chie Y., Chao-Han L. (1982). Radio wave scintillations in the ionosphere. Proc. IEEE.

[3] Shuman N. S., Hunton D. E., Viggiano A. A. (2015). Ambient
and Modified Atmospheric
Ion Chemistry: From Top to Bottom. Chem. Rev..

[4] Bernhardt P. A., Siefring C. L., Briczinski S. J., Viggiano A., Caton R. G., Pedersen T. R., Holmes J. M., Ard S., Shuman N., Groves K. M. (2017). A physics-based model for the ionization
of samarium
by the MOSC chemical releases in the upper atmosphere. Radio Sci..

[5] Holmes J. M., Dressler R. A., Pedersen T. R., Caton R. G., Miller D. (2017). A combined
spectroscopic and plasma chemical kinetic analysis of ionospheric
samarium releases. Radio Sci..

[6] Ard S. G., Shuman N. S., Martinez O., Brumbach M. T., Viggiano A. A. (2015). Kinetics
of chemi-ionization reactions of lanthanide metals (Nd, Sm) from 150
to 450 K. J. Chem. Phys..

[7] Caton R. G., Pedersen T. R., Groves K. M., Hines J., Cannon P. S., Jackson-Booth N., Parris R. T., Holmes J. M., Su Y.-J., Mishin E. V., Roddy P. A., Viggiano A. A., Shuman N. S., Ard S. G., Bernhardt P. A., Siefring C. L., Retterer J., Kudeki E., Reyes P. M. (2017). Artificial ionospheric modification:
The Metal Oxide Space Cloud experiment. Radio
Sci..

[8] Paulovič J., Gagliardi L., Dyke J. M., Hirao K. (2004). The gas-phase chemiionization
reaction between samarium and oxygen atoms: A theoretical study. J. Chem. Phys..

[9] Cox R. M., Kim J. S., Armentrout P. B., Bartlett J., Van Gundy R. A., Heaven M. C., Ard S. G., Melko J. J., Shuman N. S., Viggiano A. A. (2015). Evaluation of the exothermicity of the chemi-ionization
reaction Sm + O → SmO^+^ + *e*
^–^. J. Chem. Phys..

[10] Armentrout P. B., Cox R. M., Sweeny B. C., Ard S. G., Shuman N. S., Viggiano A. A. (2018). Lanthanides as Catalysts: Guided Ion Beam and Theoretical
Studies of Sm^+^ + COS. J. Phys. Chem.
A.

[11] Lachowicz A., Perez E. H., Shuman N. S., Ard S. G., Viggiano A. A., Armentrout P. B., Goings J. J., Sharma P., Li X., Johnson M. A. (2021). Determination
of the SmO^+^ bond energy by
threshold photodissociation of the cryogenically cooled ion. J. Chem. Phys..

[12] Ghiassee M., Kim J., Armentrout P. B. (2019). Evaluation
of the exothermicity of
the chemi-ionization reaction Nd + O → NdO^+^ + *e*
^
*‑*
^ and neodymium oxide,
carbide, dioxide, and carbonyl cation bond energies. J. Chem. Phys..

[13] Larsen M. F., Mikkelsen I. S., Meriwether J. W., Niciejewski R., Vickery K. (1989). Simultaneous observations
of neutral winds and electric
fields at spaced locations in the dawn auroral oval. J. Geophys. Res.: Space Phys..

[14] VanGundy R. A., Persinger T. D., Heaven M. C. (2019). Low energy states
of NdO^+^ probed by photoelectron spectroscopy. J. Chem.
Phys..

[15] Demireva M., Kim J., Armentrout P. B. (2016). Gadolinium (Gd) Oxide, Carbide, and
Carbonyl Cation Bond Energies and Evaluation of the Gd + O →
GdO^+^ + *e*
^
*‑*
^ Chemi-Ionization Reaction Enthalpy. J. Phys. Chem. A.

[16] Carette P., Hocquet A., Douay M., Pinchemel B. (1987). Laser-induced
fluorescence of gadolinium oxide (GdO). J. Mol.
Spectrosc..

[17] Dmitriev Y. N., Kaledin L. A., Kobylyanskii A. I., Kulikov A. N., Shenyavskaya E. A., Gurvich L. V. (1987). Electronic spectra
of diatomic molecules containing
f-elements: gadolininum monoxide, europium monofluoride, and uranium
monoxide. Acta Phys. Hung..

[18] Dmitriev Y. N., Kaledin L. A., Shenyavskaya E. A., Gurvich L. V. (1984). Electronic spectrum
of gadolinium monoxide. Acta Phys. Hung..

[19] Kaledin L. A., Erickson M. G., Heaven M. C. (1994). Laser spectroscopy
of GdO: ligand
field assignments of 4f^7^(^8^Σ)­6p ←
4f^7^(^8^Σ)­6s transitions. J. Mol. Spectrosc..

[20] Suarez C. B., Grinfeld R. (1970). Contribution to the study of gadolinium oxide band
spectrum. J. Chem. Phys..

[21] Gibson J. K. (2003). Role of
Atomic Electronics in f-Element Bond Formation: Bond Energies of Lanthanide
and Actinide Oxide Molecules. J. Phys. Chem.
A.

[22] Nakamura T., Schoendorff G., Yang D.-S., Gordon M. S. (2025). Systematic Investigation
of Electronic States and Bond Properties of LnO, LnO^+^,
LnS, and LnS^+^ (Ln = La-Lu) by Spin-Orbit Multiconfiguration
Perturbation Theory. J. Chem. Theory Comput..

[23] Wu Z. J., Guan W., Meng J., Su Z. M. (2007). Density functional
studies of diatomic LaO to LuO. J. Cluster Sci..

[24] Schaller S., Gewinner S., Schoellkopf W., Meijer G., Fielicke A. (2024). Gas-phase
vibrational spectroscopy of the dysprosium monoxide molecule and its
cation. Phys. Chem. Chem. Phys..

[25] Schaller, S. Photoionization Spectroscopy of Metal-Containing Diatomics; Technischen Universität Berlin, 2025.

[26] Willson S. P., Andrews L. (1999). Characterization of the Reaction
Products of Laser-Ablated
Early Lanthanide Metal Atoms with Molecular Oxygen. Infrared Spectra
of LnO, LnO^+^, LnO^–^, LnO_2_,
LnO_2_
^+^, LnO_2_
^–,^ LnO_3_
^–^, and (LnO)_2_ in Solid Argon. J. Phys. Chem. A.

[27] Carette P., Hocquet A. (1988). Ligand field calculation of the lower electronic energy
levels of the lanthanide monoxides. J. Mol.
Spectrosc..

[28] NIST Atomic Spectra Database. http://www.nist.gov/pml/data/asd.cfm. (accessed July 31, 2026).

[29] Pedley J. B., Marshall E. M. (1983). Thermochemical data for gaseous monoxides. J. Phys. Chem. Ref. Data.

[30] Smirnov A. N., Solomonik V. G. (2022). Accurate
spectroscopy, dipole moment, and ionization
energy of gadolinium monoxide from high-level electronic structure
calculations. Chem. Phys. Lett..

[31] Heaven M. C. (2006). Probing
actinide electronic structure using fluorescence and multi-photon
ionization spectroscopy. Phys. Chem. Chem. Phys..

[32] Heaven, M. C. ; Peterson, K. A. Probing Actinide Bonds in the Gas Phase. In Experimental and Theoretical Approaches to Actinide Chemistry; Wiley, 2018; pp 1–52 10.1002/9781119115557.ch1.

[33] Bujin G., Linton C. (1991). Examination of rotational
structure, Λ doubling,
and perturbations in 12 low lying electronic states of samarium monoxide. J. Mol. Spectrosc..

[34] Linton C., James A. M., Simard B. (1993). Stark effect measurement
in samarium
monoxide: dipole moments of the [16.6]­1 and X0^–^ states. J. Chem. Phys..

[35] Schmitz J. R., Le A. T., Steimle T. C., Rodriguez A., Heaven M. C. (2022). Electronic Spectroscopy of SmO in the 645 to 670 nm
Range. J. Phys. Chem. A.

[36] Werner H.-J., Knowles P. J., Manby F. R., Black J. A., Doll K., Heßelmann A., Kats D., Köhn A., Korona T., Kreplin D. A., Ma Q., Miller T. F., Mitrushchenkov A., Peterson K. A., Polyak I., Rauhut G., Sibaev M. (2020). The Molpro
quantum chemistry package. J. Chem. Phys..

